# From Detector Innovation to Clinical Practice: A Comprehensive Review of CZT-SPECT in Coronary Artery Disease Imaging

**DOI:** 10.2174/0115734056412716251027064220

**Published:** 2025-11-28

**Authors:** Ajay Kumar Chaudhary, Zekun Pang, Jianming Li

**Affiliations:** 1 International Medical College, Tianjin Medical University, Tianjin, China; 2 Nuclear Medicine Department, Tianjin Medical University Clinical Cardiovascular Institute, Tianjin, China; 3 Nuclear Medicine Department, Tianjin University TEDA International Cardiovascular Hospital, Tianjin, China

**Keywords:** Cadmium-zinc-telluride, Single photon emission computed tomography, Myocardial perfusion imaging, Coronary artery disease, Quantitative myocardial blood flow, Risk stratification

## Abstract

Cadmium-Zinc-Telluride (CZT) detector-based Single Photon Emission Computed Tomography (SPECT) represents a paradigm shift in Myocardial Perfusion Imaging (MPI), overcoming major limitations inherent to conventional Anger-type systems, including prolonged acquisition times and constrained quantitative functionality. The cardiac-optimized CZT platform enables rapid image acquisition with reduced radiation burden while achieving enhanced diagnostic precision through superior spatial resolution and photon sensitivity. Clinical evidence demonstrates superior performance in detecting hemodynamically significant Coronary Artery Disease (CAD) compared to traditional SPECT, coupled with quantitative assessment of myocardial blood flow and flow reserve, which strengthens risk stratification and prognostic capability. This technology supports personalized clinical management through improved detection of subclinical ischemia and protocol optimization for radiation reduction. Integration with advanced attenuation/scatter correction methodologies enhances prognostic discrimination, enabling robust differentiation between low-risk and high-risk patient cohorts for Major Adverse Cardiac Events (MACEs). Persistent challenges, including motion-related artifacts and protocol standardization, are being addressed through innovations in data-driven motion correction, next-generation detector architectures, collimators, and hybrid imaging system integration. As the field of cardiovascular imaging evolves, CZT-SPECT stands as a transformative modality that optimally balances operational efficiency, patient safety, and diagnostic confidence. Continued technological refinement and rigorous clinical validation will solidify its position as an indispensable tool for guiding precision interventions and optimizing CAD management pathways.

## 1 INTRODUCTION

The evolution of Single-Photon Emission Computed Tomography (SPECT) over the decades has significantly impacted nuclear cardiology in the diagnosis of known or suspected Coronary Artery Disease (CAD). In the early days of SPECT, scintillation crystals, specifically NaI crystals, were used; however, clinicians faced significant limitations in speed and image quality, relatively high radiation exposure for patients, and quantification ability for myocardial flow. The introduction of cardiac-dedicated CZT-based detectors in SPECT has been a game-changer in the field of nuclear cardiology. CZT detectors are based on solid-state detectors that directly convert gamma photons into electrical signals, eliminating the need for photomultiplier tubes, and enhancing energy resolution (2-3 mm spatial resolution) and sensitivity (up to 5-10× higher than NaI systems). This technological leap enables significant reductions in scan time (from 15-20 minutes to 2-5 minutes), radiopharmaceutical doses (by 50%-70%), and radiation exposure while improving image quality. Clinically, cardiac-dedicated CZT-SPECT has demonstrated superior diagnostic accuracy in MPI, particularly for detecting CAD as well as the ability to quantify Myocardial Blood Flow (MBF) and Myocardial Flow Reserve (MFR), which delivers promising results in diagnostic efficiency and prognostic evaluation for CAD, ultimately leading to better patient management and outcomes. This article will discuss the latest progress in techniques and diagnosing CAD with cardiac-dedicated CZT-SPECT MPI from the following aspects.

## 2 MATERIALS AND METHODS

In this study, we conducted a comprehensive literature review utilizing several resources, including Springer Nature, Elsevier, PMC, and ScienceDirect. Our review studies had analyzed literature from the year 2004 to 2025. The search terms are CZT-SPECT, cadmium-zinc-telluride, myocardial perfusion imaging, diagnostic accuracy, and myocardial flow reserves. This review primarily examines the use of CZT SPECT technology for myocardial perfusion imaging in evaluating coronary artery disease, compares the advancement of CZT technology over conventional SPECT, and assesses its prognostic and functional capabilities.

## 3 CZT TECHNOLOGY

The development of Cadmium-Zinc-Telluride (CZT) detector technology marks a significant advancement in nuclear cardiology, especially for Myocardial Perfusion Imaging (MPI) with the commercialization of CZT-based cardiac SPECT cameras. Additionally, the limitations of conventional sodium iodide (NaI)-based Anger cameras become more apparent, particularly regarding resolution, count sensitivity, and acquisition time. However, solid-state detectors like CZT have emerged as a transformative alternative, revolutionizing nuclear cardiology.

### 3.1 Evolution of CZT Technology

The initial exploration of solid-state semiconductors for gamma-ray detection began in the early 1980s, with researchers investigating materials like HgI_2_ and CdTe [1]. CZT, a cadmium telluride derivative doped with zinc, offers enhanced room-temperature performance and superior energy resolution. However, several efforts have been made to improve the crystal growth and pixilation of CZT for imaging applications [2].

By the early 2000s, CZT arrays were tested for small-field applications, including small animal and breast imaging. The turning point occurred with the development of dedicated cardiac CZT-SPECT systems. GE Healthcare launched the Discovery NM 530c in 2009, the first FDA-approved solid-state cardiac-dedicated SPECT system [3]. Around the same time, Spectrum Dynamics introduced the D-SPECT system, which featured rotating column detectors and high temporal resolution; these innovations laid the groundwork for the clinical use of CZT in MPI.

### 3.2 Principles of CZT Detectors

CZT is a direct-conversion semiconductor detector that differs fundamentally from traditional NaI scintillation detectors. In CZT, incident gamma photons are directly converted into electrical signals. This allows for the elimination of intermediate scintillation and photomultiplier tube stages, resulting in improved energy resolution [2, 3].

CZT detectors typically operate with pixelated arrays, where each pixel functions as an individual detector. Furthermore, narrower energy peak windows during acquisition significantly reduce scatter and parallax errors, which in turn enhance spatial resolution. CZT systems also maintain good linearity across energy levels, making them suitable for multi-isotope imaging.

Additionally, because CdZnTe crystals have high density (~5.8g/cm^3^) as well as high atomic number (48, 30, 52) can directly convert gamma photons into electrical signals at room temperature without the need for a photomultiplier tube, and also simplifies the probe structure and saves space, and also avoids the loss and distortion of the signal conversion process to a certain extent, thus increasing the energy resolution as well as the separation of spectral peaks.

### 3.3 System Design and Imaging Geometry

CZT cameras differ from traditional SPECT systems not only in their detector material but also in camera design. Systems like the GE Discovery NM/CT 530c and NM 570c utilize multiple stationary detectors arranged in a curved geometry, enabling complete cardiac coverage without the need for mechanical rotation. Alternatively, the Spectrum Dynamics D-SPECT system utilizes nine independent rotating columns equipped with wide-angle collimators and high-sensitivity tracking, enabling dynamic acquisition and high count rates.

The Discovery NM 530c and Discovery NM/CT 570c (which add a CT unit to the Discovery NM 530c while keeping the same components) feature multi-hole (19-hole) collimators, each with a 4.75 mm aperture and fixed-angle acquisition, improving spatial resolution while maintaining high count activity. The detector is a square unit measuring 4 cm by 4 cm, with a thickness of 5 mm, and pixel sizes of 2.46 mm by 2.56 mm. Each detector array contains four units, each with elements measuring 2.56 mm by 2.56 mm by 5 mm, arranged in a 16 by 16 element grid. Out of the 19 pinhole collimators, 9 are oriented perpendicular to the patient's long axis, while 5 are angled above, and 5 below the axis to enable three-dimensional acquisition. The detector module is mounted on a gantry that allows for patient positioning in both supine and prone positions. This design provides consistent angular data and preserves sensitivity for dynamic studies [4, 5].

Unlike the Discovery NM 530c, D-SPECT offers a different design and is produced by Spectrum Dynamics. The D-SPECT detector has 1024 CZT elements (2.56mm by 2.56mm by 5mm) arranged in a 16 by 64 array. It uses parallel-hole collimators with CZT detectors, enabling 120-degree scans focused on cardiac regions. A notable feature is the flexible examination position, allowing patients to sit or lie down; the seated position offers greater comfort [3, 5].

### 3.4 Validation of CZT SPECT

In the early days, for the validation of CZT-SPECT (Discovery NM 530c, and D-SPECT), several single-center and multi-center trial studies have been done, and the result was that the time of acquisition was decreased by 3-5 fold as compared to conventional SPECT long while maintaining similar image quality and diagnostic performance [4, 6, 7]. A CZT-SPECT study shows that the dual-isotope stress ^201^Ti/rest ^99m^Tc protocol can be performed in 20 minutes with image quality and dose similar to those of conventional SPECT [3, 8]. However, a study of Askew JW. *et al*.,’s findings does not support the routine use of early image acquisition with this new solid-state ultra-fast camera system [9].

## 4 ADVANCES OF CZT-SPECT IN IMAGE ACQUISITION, RECONSTRUCTION, COLLIMATION, AND CORRECTION TECHNIQUES

Significant advances have been made across different key domains for CZT-SPECT in MPI. Several new protocols were being explored to improve procedure efficiency and image quality. An MPI study by Mouden M. *et al*., demonstrated the robustness of the CZT camera compared to conventional SPECT (C-SPECT), showing that the CZT camera reduced rest imaging by 56% (vs. 35% with C-SPECT), lowered the average isotope dose administered (658 ± 390 *vs*. 840 ± 421 MBq), and shortened imaging times (6.39 ± 1.91 *vs*. 20.40 ± 7.46 minutes) [10]. Case JA., *et al*., evaluated a D-SPECT system with CT attenuation correction using an early post-injection, stress-first SPECT protocol to replace standard delayed imaging. Compared to standard imaging, early post-injection (17 ± 2 minutes) achieved comparable image quality, higher reader confidence (89.5% agreement), and reduced total imaging time (60 ± 18 *vs*. 104 ± standard minutes) [11]. Mochula A., *et al*., reported that a one-compartment kinetic model combined with attenuation correction increased stress/rest MBF and flow differences compared to non-attenuation correction [12]. Zhao F. *et al*., highlighted that CTAC enhances the assessment of viable myocardium for decision-making in revascularization therapy [13]. Moreover, the novel emission-based attenuation correction methods, known as CASCTEC-AC or CT-free, developed by Hesse M. *et al*., utilize tissue segmentation to generate attenuation maps [14]. In a related clinical trial study by Sprauel T., *et al*., which focused on Deep Learning Attenuation Correction (DLAC *vs*. PET), similar findings were reported [15]. These results highlighted improved tracer homogeneity, maintained accuracy in detecting perfusion defects compared with SPECT/CT-AC, and enhanced uptake in inferior myocardial segments in comparison to both SPECT/CT-AC and PET [14, 15]. These studies suggest promising advancements in the reduction of acquisition time as well as optimization of tracer dose using CZT-SPECT and different attenuation correction techniques with and without CTAC.

Daou D. *et al*., utilized data-driven cardiac respiratory motion correction to compare its feasibility and performance with ECG monitor-based triggers for evaluating MPI studies. This revealed near-perfect agreement between cardiac contraction triggers generated by both methods, with data-driven techniques providing comparable and well-correlated LV global systolic parameters and similar cine image quality at stress and rest [16]. The Post-reconstruction Motion Correction with Optical Flow Estimation (PMC-OFE) by Song C., *et al*., achieved significant detection of perfusion defects across reduced-dose SPECT imaging, with AUC values surpassing traditional methods (0.813 at half dose *versus* 0.795 for standard dose Non-MC). Additionally, it highlights PMC-OFE's potential to maintain diagnostic accuracy at a 75-87.5% dose reduction, outperforming existing methods, such as “Motion Frozen” (AUC: 0.779 *vs*. 0.731 at a 12.5% dose) [17].

In 2016, Weng F. *et al*., pioneered the development of an energy-optimized parallel hole collimator for CZT detectors, using Geant4 simulations to balance sensitivity and resolution across multiple radiotracers (^57^Co, ^99m^Tc, ^123^I, ^111^I). This work demonstrated that a square-hole design (23mm length, 1.28mm width, 0.32mm septal thickness) could match or exceed commercial collimators (LEHR/MEGP), addressing a critical need to reduce clinical workflow disruptions caused by frequent collimator swaps [18]. Santarelli MF., *et al*., compared dedicated cardiac (Alcyone) *vs*. general-purpose (Discovery NM/CT 670 CZT, GE Healthcare) SPECT systems with collimators, revealing that organ-specific designs excel in cardiac imaging (superior SBNR/TBCNR), but lack versatility for broader applications or high BMI patients [19]. In the year 2024, a study by Ghoneim MA revealed the optimal diameter of the pinhole collimator was 1.38 ± 0.081mm to reconcile the trade-offs between sensitivity and resolution [20], while the study of Vauchot F., *et al*., highlighted that the Butterworth filter optimization study reduced MPI artefacts by adjusting cutoff frequencies (25% *vs*. 37% Nyquist), improving specificity (69.7%±16.2% artifact reduction) without compromising sensitivity in CZT-SPECT [21].

However, multiple innovations have been made to optimize diagnostic workflows while minimizing patient radiation exposure and imaging time, balancing the sensitivity and diagnostic performance. The primary trend was a shift toward faster, lower-dose, and more precise examinations. New protocols are significantly reducing imaging times, while advanced software for attenuation and motion correction is boosting diagnostic accuracy even at lower tracer doses. Additionally, hardware improvements, especially in collimator design, have enhanced the fundamental balance between resolution and sensitivity. Although this approach shows promise for improving CAD diagnostic accuracy through optimized MPI interpretation, clinical implementation requires standardization and broader validation.

## 5 COMPARISON OF CARDIAC-DEDICATED CZT-SPECT WITH C-SPECT IN ROUTINE MPI FOR DIAGNOSING CAD

CAD is a prevalent condition with a significant impact on myocardial perfusion. Yokota S. *et al*., performed a study involving 256 patients with routine MPI who underwent invasive angiography due to persistent or worsening symptoms; 36% were found to have significant CAD. Among these, significant Left Main (LM) disease was present in 7%, three-vessel disease in 10%, two-vessel disease in 22%, and single-vessel disease (not left main) in 61%. In single-vessel disease, the location breakdown was the Left Anterior Descending (LAD) in 40%, the Right Coronary Artery (RCA) in 30%, and the Left Circumflex (LCX) in 30%. This shows that a substantial portion of patients with seemingly normal MPI may have underlying CAD, suggesting limitations in current perfusion imaging techniques and the need for improved diagnostic accuracy [22].

Several aspects need to be considered when comparing CZT-SPECT with C-SPECT for myocardial imaging in terms of diagnostic performance, where a meta-analysis study by Cantoni V., *et al*., revealed modest superiority in diagnostic performance for CZT-SPECT (sensitivity:89% *vs*. 85%; specificity: 69% and 66%). The slightly higher AUC (0.89 *vs*. 0.83) suggests that CZT-SPECT may better detect angiographically proven CAD [23]. Similarly, to further validate the CZT-SPECT, Kalantari F. *et al*., confirmed excellent inter- and intra-observer reader agreement (Kappa= 0.87-0.93) and a trend toward better sensitivity, PPV, NPV, and accuracy with CZT-SPECT, though not statistically significant [24] (Table **1**). However, marginal gains in sensitivity and specificity or statistically insignificant results indicate that neither modality was flawless; challenges remain for validation.

CAD drives distinct pathophysiological alterations in myocardial perfusion dynamics. De Luca G., *et al*., studied in Egypt, and the analysis revealed that multivessel CAD occurs in 58.2% of ST elevation myocardial infarction patients undergoing primary percutaneous coronary intervention. CAD severity independently correlated with impaired angiographic perfusion (adjusted OR 1.18, 95% CI 1.01–1.40; *p* = 0.049) and elevated mortality risk (adjusted HR 1.54, 95% CI 1.06–2.24; *p* = 0.022) during follow-up (208 ± 160 days). These observations underscore the prognostic significance of characterizing CAD-mediated perfusion abnormalities for optimizing clinical management [25].

Diagnostic performance evaluation must account for imaging parameters beyond accuracy. Wu D., *et al*., highlight operational advantages of CZT-SPECT, including faster acquisition and comparable assessment of left ventricular scar, functional indices, and dyssynchrony metrics in heart failure patients. Notably, CZT-SPECT exhibited systematic underestimation of Summed Rest Scores (SRS) and Total Perfusion Defects (TPD) compared to conventional systems, while overestimating phase histogram Bandwidth (BW) and Standard Deviation (SD) in mechanical synchrony analysis. Such modality-specific variations necessitate careful interpretation of quantitative parameters [26]. The enhanced operational efficiency positions CZT-SPECT as a pragmatic option for multidimensional myocardial evaluation in heart failure management.

Overall, while CZT-SPECT enhances efficiency and reproducibility and provides a modest improvement in diagnostic accuracy, its clinical significance is rooted in facilitating a more thorough and practical evaluation of CAD-related perfusion abnormalities. Nonetheless, challenges persist in identifying diffuse ischemia and validating quantitative indices across various patient populations.

## 6 QUANTITATIVE MPI WITH CARDIAC-DEDICATED CZT-SPECT

### 6.1 Pathophysiological Insights from Cardiac-dedicated CZT-SPECT in MPI

Coronary Artery Disease (CAD) is a common condition that significantly impacts myocardial perfusion. It can cause various pathophysiological changes in how blood flows through the heart muscle. DeLuca G., *et al*., reported that the severity of CAD has been independently linked to impaired angiographic myocardial perfusion and higher mortality over an average follow-up of 208 ± 160 days [25]. Additionally, Yokota S. *et al*., show that among patients with normal Myocardial Perfusion Imaging (MPI) who underwent invasive angiography due to ongoing or worsening symptoms, 36% were found to have significant CAD [22]. The above studies highlighted the importance of understanding CAD’s prevalence, acknowledged limitations in diagnosing CAD, and emphasized the need to address these challenges to improve patient care and outcomes.

Quantitative MPI with CZT-SPECT operates according to fundamental principles, enabling measurement of critical parameters including MBF, MFR, and Relative Flow Reserve (RFR), which offer crucial insights into CAD pathophysiology. The assessment of MBF and MFR demonstrates clinical utility for detecting CAD presence and severity while overcoming limitations related to the distribution patterns of radioactive tracers in myocardial tissue, thereby enhancing diagnostic accuracy by preventing oversight of balanced ischemia. Wang J. *et al*., conducted participant-level analysis of 49 patients with suspected/known CAD undergoing both CZT-SPECT and coronary angiography (using ≥ 50% stenosis threshold), quantitative *vs*. semi-quantitative assessment of MBF and MFR demonstrated superior sensitivity, specificity, positive predictive value, negative predictive value, and accuracy compared to semi-quantitative approaches, while Receiver Operating Characteristic (ROC) analysis established optimal diagnostic thresholds for ≥ 50% and 75% stenosis stress MBF (1.61 and 1.15ml/min/g) and MFR (1.86 and 1.71), confirming their diagnostic validity [27].

Further validation came from Pang Z. *et al*., a vessel-level investigation of 57 consecutive patients with suspected/known CAD undergoing low-dose dynamic CZT-SPECT and angiography revealed comparable resting MBF (rMBF) between negative and positive groups (*p* > 0.05) when using either ≥ 50% or ≥ 75% angiographic stenosis thresholds. However, significant reductions in sMBF and MFR were observed in positive groups (all *p* < 0.05), with strong correlations emerging between sMBF-MFR, sMBF-RFR, and MFR-RFR (all *p* < 0.0001), indicating parameter interdependence for CAD diagnosis [28]. Larger studies by Acampa W. *et al*., (n = 173) confirmed these findings, showing markedly depressed hyperemic MBF (2.59 *vs*.3.24 ml/min/g) and MFR (1.96 *vs*. 2.74) in patients with obstructive CAD, underscoring CZT-SPECT's capability in characterizing obstructive disease and perfusion abnormalities for clinical decision-making [29].

Importantly, the feasibility study carried out by Mesquita C. *et al*., evaluated cardiac-dedicated D-SPECT for MFR assessment using Cedars-Sinai QPS kinetic software (abnormal MFR threshold < 2). While 13 patients exhibited global MFR < 2, 11 demonstrated regional MFR reductions despite preserved global values. Notably, six patients with low MFR showed no SPECT perfusion defects. Significant LVEF reductions were observed in MFR< 2 *versus* MFR ≥ 2 groups, confirming CZT-SPECT's utility in identifying impaired MFR for optimized CAD management [30].

Together, these studies demonstrate CZT-SPECT's enhanced capability for comprehensive CAD assessment through quantitative perfusion analysis, offering improved detection of balanced ischemia and early perfusion changes that conventional methods might miss. Nonetheless, all these studies show robust CZT performance, but the threshold values of MBF and MFR varied across the studies. Therefore, for the standardization of the threshold value, a more diverse population study is needed.

### 6.2 Validation against PET, Invasive Angiography, and Cardiac MRI

The validation of CZT-SPECT for MPI has progressed through distinct phases over 15 years. Nkoulou R., *et al*., reported that rest MBF was comparable between CZT-SPECT and PET (IQR: 0.89 *vs*. 0.92 ml/g/min, *p* = NS), while stress MBF was significantly lower with CZT (IQR: 1.11 *vs*. 2.06 ml/g/min, *p* < 0.001), resulting in a lower MFR (1.32 *vs*. 2.36; *p* < 0.001) and an MFR cutoff of 1.26 on CZT predicted abnormal PET values with 75% accuracy [31]. Diagnostic validation, as demonstrated by Agostini D. *et al*., showed that CZT yielded higher MBF than PET at rest and stress for global, LAD, and LCX territories (but not RCA), while MFR was similar across modalities. Diagnostic performance for detecting ischemia was strong, with sensitivity, specificity, accuracy, PPV, and NPV (83.3%, 95.8%, 100%, and 85.7%); and for identifying significant stenosis (FFR ≤ 0.8), sensitivity, specificity, and accuracy were 58.3%, 84.6%, and 81.1% [32].

A study by Otaki Y. *et al*., highlights subsequent technical refinement strengthened correlations (global MBF r = 0.91, regional r = 0.86; *p* < 0.001) while RCA specific limitations (rest MBF 0.81 *vs*. 0.98, *p* = 0.03) remained [33]. Li C., *et al*., and He M, *et al*., established functional correlations with CFR–FFR (r = 505, *p* = 0.003), CFR-TIMI frame count (LAD r = 0.506, LCX r = -0.532, and RCA r = -0.657), determining vessel-specific CFR cut-offs (1.73-2.47) with up to 100% LAD sensitivity [34, 35].

Recently, Yahiro DS *et al*., have conducted a meta-analysis of 268 patients, confirming minimal bias (rest MBF difference, 0.006 ml/min/g) while maintaining 80.8% sensitivity/ 87% specificity compared to PET [36]. A parallel clinical study by Sano A. *et al*., demonstrated exceptional viability prediction (AUC 0.97) and a 7.1% ± 5% absolute LVEF improvement post-revascularization *versus* medical therapy (*p* = 0.008) [37]. Throughout this evolution, excellent reader agreement (k=0.87-0.93; Kalantari F *et al*.,) and operational advantages, such as faster acquisition time [24], were confirmed.

Collectively, these studies confirm the clinical viability of CZT-SPECT for quantitative perfusion and flow assessment, demonstrating comparable diagnostic performance to PET and FFR across multiple territories. However, discrepancies in stress MBF across modalities, particularly in RCA territories, reflect known limitations in detector geometry and temporal resolution. Despite this, recent meta-analysis and dynamic flow studies demonstrate strong correlation, reproducibility, and prognostic value, indicating CZT-SPECT's growing potential as a non-invasive alternative in hybrid or standalone cardiac workflows.

### 6.3 Risk Stratification in MPI Using CZT-SPECT

Risk stratification in MPI using CZT-SPECT is crucial for patient management and prognostic outcomes. Computed Tomography Attenuation Correction (CTAC) can improve the risk stratification accuracy of MPI. Ou X. *et al*., conducted a study of 935 patients who underwent Technetium-99m Sestamibi stress/rest MPI with SPECT and CTAC, at the end of a mean follow-up period of 2.2 ± 0.8 years, the annual frequency of Major Adverse Cardiac Events (MACEs) was 0.5% in patients with normal Summed Stress Score (SSS), 2% in those with mildly abnormal SSS, and 8% in those with moderately or severely abnormal SSS. With CTAC, more studies were categorized as normal, and the number of patients with moderately to severely abnormal perfusion on CTAC was reduced. The annual frequency of MACEs increased significantly after CTAC in the moderately to severely abnormal group (4.5 *vs*. 8.1%, *p* = 0.03), indicating improved risk stratification [38]. Klein E. *et al*., reported similar findings, as did Feher A. *et al*., in a study that focused on obese patients. The combination of CZT-SPECT and attenuation correction demonstrates robust performance for risk stratification and MACEs prediction, while combining with stress-Total Perfusion Deficit (TPD) provides additional prognostic accuracy [39, 40].

MFR measured by CZT-SPECT has prognostic value for risk stratification in patients. Li L. *et al*., conducted a study of 303 patients with non-obstructive CAD (ischemia with non-obstructive coronary artery, INOCA) and obstructive CAD (OCAD). Kaplan-Meier survival curves showed that the incidence of MACE was similar in the INOCA and OCAD groups (log-rank *p* = 0.2645), but those with reduced MFR had a higher incidence of MACE than those with normal MFR (log-rank *p* = 0.0019). Univariable Cox regression analysis showed that for every 1-unit increase in MFR, the risk of MACE for INOCA was reduced by 66.1% and that for OCAD by 64.2%. These results suggest that MFR measured by CZT-SPECT can help stratify patients according to their risk of MACE, enabling more personalized treatment plans [41].

## 7 ROLE OF CZT-SPECT IN THERAPEUTIC STRATEGIES AND CLINICAL PRACTICE

### 7.1 Guiding Therapeutic Interventions

CZT-SPECT serves as a crucial modality for guiding therapeutic decision-making while providing quantitative assessment and prognostic stratification in patients with INOCA. Zhang H., *et al*., performed a study involving 118 INOCA patients undergoing CZT-SPECT imaging with concurrent invasive coronary angiography, which demonstrated that during a median 15-month follow-up period, both stress Myocardial Blood Flow (sMBF) and Coronary Flow Reserve (CFR) were significantly reduced in patients experiencing Major Adverse Cardiovascular Events (MACEs). The established optimal prognostic thresholds of sMBF < 3.16 and CFR < 2.52 revealed both impaired sMBF (HR: 15.08; 95% CI 2.95-77.07; *p* < 0.001) and reduced CFR (HR: 6.51; 95% CI 1.43-29.65; *p* < 0.001) as independent predictors of MACEs [42]. These findings, by Li L. *et al*., and Zhang H. *et al*., enable clinicians to inform clinical risk stratification and the occurrence of MACEs, and to guide early preventive measures and targeted interventions [41, 42].

Beyond prognostic stratification, CZT-SPECT is useful for evaluating revascularization requirements. A comparative analysis by Van Dijk JD. *et al*., demonstrated that a standard Fixed-Activity Protocol (FAP) *versus* a Patient-specific Low-Activity Protocol (PLAP) in CZT-SPECT myocardial perfusion imaging revealed comparable rates of normal scan interpretations and similar prognostic outcomes between the two groups, with PLAP significantly reducing mean radiation exposure [43]. This evidence positions CZT-SPECT as a dual-purpose modality that not only guides therapeutic interventions but also optimizes radiation safety parameters—a critical consideration for enhancing patient safety and minimizing long-term radiation-associated risks [43].

### 7.2 Impact of Quantitative MPI on Patient Management

Quantitative MPI has a significant impact on invasive patient management. Promteangtrong C. *et al*., conducted a study with 57 patients who underwent [^15^O] H_2_O PET/CT MPI; the referral rate for invasive CAG and revascularization was significantly different between the normal and abnormal MPI groups. The referral rate was 5.6% in the normal group and 48.7% in the abnormal group (*p* = 0.002), whereas hyperemic MBF was a significant predictor for referral (OR 15.24, 95% CI 3.39 - 68.55, *p* < 0.005) and revascularization (OR 28.57, 95% CI 3.08 - 265.33, *p* < 0.005) [44]. This study's results are similar to those of Zhang H. *et al*., who found that quantitative MPI with decreased hyperemic MBF or sMBF can assist clinicians in making more informed decisions about invasive procedures for managing CAD or predicting MACEs [44, 42].

Automated quantitative viability assessment *via* Myocardial Perfusion Imaging (MPI) demonstrates prognostic utility in predicting survival outcomes in patients with Ischemic Cardiomyopathy (ICM). A cohort study by Hage FG. *et al*., involved 246 consecutive ICM patients undergoing rest-redistribution gated SPECT thallium-201 MPI demonstrated superior survival outcomes with Coronary Revascularization (CR) *versus* Medical Therapy (MT) [45]. Survival benefits from CR showed an inverse correlation with non-viable myocardial burden (5% mortality risk elevation per 1% increase in non-viable myocardium extent, *p* = 0.009). These observations indicate that quantitative MPI viability analysis offers critical prognostic stratification to inform clinical decision-making between revascularization and conservative management.

In Heart Failure (HF) populations, Cadmium-Zinc-Telluride SPECT (CZT-SPECT) enables concurrent evaluation of myocardial perfusion-adrenergic innervation relationships. A study by Assante R. *et al*., investigating dual-isotope ^123^I/^99m^Tc CZT-SPECT in 36 HF patients revealed left ventricular Ejection Fraction (EF) maintained significant associations with perfusion defect extent (*p* < 0.005), sympathetic denervation severity (*p* < 0.005), and both early/late-phase ^123^I-metaiodobenzylguanidine (MIBG) parameters, including Heart-to-Mediastinum (H/M) ratios [46]. Notably, while perfusion-innervation defect sizes correlated significantly, sympathetic denervation demonstrated a stronger prognostic association than perfusion abnormalities (*p* < 0.001). This evidence positions CZT-SPECT as a multimodal imaging tool for deciphering HF pathophysiology and optimizing therapeutic targeting.

Expanding these applications, a multicenter trial was conducted by Yoshida S. *et al*., evaluating CZT-SPECT with ^99m^Tc-sestamibi for Myocardial Flow Reserve (MFR) quantification in 127 heart failure patients with preserved Ejection Fraction (HFpEF) in patients. HFpEF cohorts exhibited significantly impaired MFR *versus* non-HFpEF controls (diagnostic threshold: 2.525, AUC = 0.85) [47]. Reduced MFR values showed significant correlations with increased hospitalization rates and enhanced adverse event prediction, establishing its role in HFpEF severity stratification and prognostic modeling.

### 7.3 Integration of CZT-SPECT in Routine Clinical Practice

Several factors have facilitated the integration of CZT-SPECT in routine clinical nuclear cardiology practice. A study by D’estanque E. *et al*., for optimizing a simultaneous dual-isotope ^201^Tl/^123^I-MIBG myocardial SPECT imaging protocol with a CZT camera for trigger zone assessment after myocardial infarction, found that delayed acquisitions and scatter-corrected isotope ^201^Tl/^123^I-MIBG SPECT acquisitions provide an improved evaluation of trigger zones in routine clinical settings after revascularization for STEMI [48]. This shows that CZT-SPECT can be effectively used in specific clinical scenarios, such as post-myocardial infarction assessment.

Claudin M. *et al*., performed a study on left ventricular function assessment, with 76 patients who underwent cardiac MRI and an MPI on a CZT camera with a low-dose protocol, found that the assessment of Left Ventricular (LV) function on a CZT camera with low injected activities and limited recording times correlated well with the reference assessment from cardiac MRI. Furthermore, the correlations for ejection fraction, end-diastolic and end-systolic volumes, and segmental contractility were excellent. Additionally, the results suggest that CZT-SPECT can be routinely used for LV function assessment in clinical practice, providing reliable information with reduced radiation exposure and shorter acquisition times [49].

## 8 CONTROVERSIES AND CHALLENGES IN CZT-SPECT MPI

### 8.1 Limitations and Challenges in CZT-SPECT Technology

Despite its advantages, CZT-SPECT technology faces several technical limitations and clinical challenges, with suboptimal specificity remaining a particular concern. A systematic review and meta-analysis by Nudi F. *et al*., evaluating the diagnostic accuracy of MPI using CZT technology *versus* invasive CAG, demonstrated that while CZT-MPI achieved a sensitivity of 0.84 (95% confidence interval [CI]: 0.78 to 0.89), its specificity measured 0.69 (95% CI: 0.62 to 0.76), a result notably lower than conventional systems [50]. However, in contrast to these findings, a systematic and meta-analysis study done by Panjer M., *et al*., has found that the dynamic CZT-SPECT MPI demonstrated a good sensitivity of 0.79(95%CI 0.73-0.85) and specificity of 0.85(95%CI 0.74-0.92) to diagnose CAD as compared to the gold standard PET [51]. However, there were contrasting findings between the specificity of the two studies, which raised further clinical concern, as it may produce false-positive outcomes that could lead to unnecessary diagnostic procedures or therapeutic interventions.

Furthermore, several studies show that the threshold values for MBF and MFR vary across studies [27, 42], and two studies highlight the RCA-specific limitations with CZT [32, 33]. These discrepancies raise questions about the standardization of MBF and MFR threshold values. However, data-driven attenuation correction and DLAC overcome the limitations of RCA [14, 15]. Nonetheless, they still lack standardization and generalizability in high-BMI and female breast attenuation scenarios.

Van Dijk JD. *et al*., conducted a study that encountered a challenge: identifying patient-related factors that affect image quality. However, minimizing patient-specific tracer dose in MPI using CZT-SPECT revealed that in heavier patients, image quality and photon counts decreased when a fixed tracer dose was used [52]. In addition, a formula based on body weight was derived to optimize the tracer dose and scan time, but it still indicates that patient physical characteristics can affect the performance of CZT-SPECT. Additionally, motion during imaging can cause artifacts in the image. For instance, Kennedy JA. *et al*., studied a dedicated cardiac solid-state CZT-SPECT device, and motion during MPI was shown to cause clinically significant artifacts. Although a method to detect and mitigate motion was proposed, it still highlights the need for careful patient positioning and motion management during CZT-SPECT imaging [53]. Furthermore, Oddstig J. *et al*., support the notion that attenuation and injection-related artifacts are significant, as their study compared CZT with a conventional gamma camera and found that attenuation artifacts shifted from the inferolateral wall (conventional) to the lateral wall (CZT) in both patient and phantom studies. The extent of the attenuation artifact was significantly higher with the CZT camera (patients: 23 ± 5% *vs*. 15 ± 5%, *p* < 0.001; phantom: 28% *vs*. 19%). These differences underscore the need for caution when interpreting perfusion defects, as artifact location and intensity may vary between camera types [54].

Substantial heterogeneity in findings was attributed to hardware configuration (Discovery NM 530c and D-SPECT), collimator design, spatial resolution, count sensitivity, and patient positioning. D-SPECT provides better diagnostic performance in morbidly obese patients compared to Discovery NM 530c [55].

### 8.2 Debates on the Clinical Utility of Quantitative MPI

There are ongoing debates regarding the clinical utility of quantitative MPI due to the lack of standardization of acquisition protocols and tracer kinetic model selection, which complicates the comparison of different studies and modalities. A review study of Renaud JM., *et al*., evaluates the precision of SPECT-derived MFR compared to PET MFR for clinical patient categorization. Despite technological advances enabling SPECT MFR quantification, significant imprecision was observed, with correct patient classification achieved in only up to 34% of cases within typical MFR ranges (1.5-2.0) [56]. The authors conclude that current SPECT MFR estimates lack sufficient precision and require further optimization before routine clinical application for risk stratification. Additionally, the complexity of the analysis and the need for specialized software and expertise may limit the widespread adoption of quantitative MPI in routine clinical practice.

There was variability in clinical protocols among vendors, which does not indicate clinical superiority. A recent study by Wieting W. *et al*., found that using one-day protocols and attenuation correction consistently alters quantitative results, resulting in notably higher rest MBF (1.2 ± 0.5 *vs*. 1.0 ± 0.46 ml/min/g; *p* = 0.009) and consequently a lower MFR [57]. This study gives additional debates on 1-day protocols for MFR values.

While motion correction techniques (whether software-based, deep learning, or AI) and attenuation correction methods (CT or non-CT) enhance the prognostic and diagnostic value in nuclear cardiology by improving sensitivity, specificity, and balancing resolution, there are still challenges to address. Early clinical experiences with emission-based data-driven PMC-OFE and DLAC techniques have shown improvements in specificity and tracer uniformity distribution, which eliminates the need for dual scans and supports low-dose, rapid imaging [14-17]. However, concerns persist regarding the validation of these methods across different camera vendors, the cost of integration, the risk of over- or under-correcting true defects, and their generalizability in populations with high BMI, female breast considerations, and other clinically complex scenarios.

Cost-effectiveness and clinical adoption continue to be significant sources of hesitancy in clinical practice, especially in settings with lower nuclear cardiac throughput, such as community-level hospitals or developing countries. Nevertheless, C-SPECT remains globally dominant, with CZT and PET outpacing it. This trend reflects considerations of access and economics, as well as the versatility of traditional systems for a wider range of applications.

## 9 FUTURE PERSPECTIVES IN CZT-SPECT MPI

### 9.1 Future Directions and Research Needs in CZT-SPECT Imaging

Emerging advancements in CZT-SPECT imaging aim to address current technological constraints through targeted innovation. A key priority lies in optimizing the modality's diagnostic specificity through refined computational methodologies. Future studies should prioritize the development of advanced algorithms and novel techniques to mitigate false-positive interpretations, particularly through systematic investigation of scatter correction and attenuation compensation enhancements. For instance, a recent investigation evaluating a full-ring CZT-SPECT configuration for whole-body applications successfully implemented an optimized dual-energy-window scatter correction protocol specifically tailored for CZT detectors, demonstrating superior contrast-recovery performance relative to conventional correction approaches [58]. Such innovations warrant rigorous exploration in myocardial perfusion imaging protocols to optimize diagnostic precision. Additionally, standardizing quantitative flow protocols across vendors and platforms is necessary to enhance reproducibility and comparability. Furthermore, long-term prognostic validation in diverse large populations beyond CAD, including heart failure and microvascular dysfunction, needs to be addressed. This includes the high cost, which necessitates adequate studies in larger populations to justify adoption in the community-level healthcare systems in developing countries. The integration of hybrid modalities, along with AI integration studies, should be conducted in multi-center studies.

In MPI, continued research could lead to better-quality images, allowing for more accurate detection of perfusion defects. Additionally, research on reducing the impact of patient-related factors such as motion and body size on image quality is needed. Developing more effective motion correction techniques and patient-tailored acquisition protocols could enhance the overall performance of CZT-SPECT in clinical settings.

### 9.2 Emerging Trends and Innovations in CZT-SPECT Technology

Emerging trends in CZT-SPECT technology are focused on improving its performance and expanding its applications. The data-driven gating techniques were innovative techniques that demonstrated the feasibility of a data-driven cardiac Respiratory Motion (RM) correction method (REGAT) in CZT-SPECT MPI [16]. Additionally, this technique showed almost perfect agreement between data-driven and ECG monitor-based cardiac contraction triggers, providing comparable LV global systolic function parameters and cine image quality. Such advancements could improve the accuracy of functional assessments of MPI study procedures.

Concurrent development involves advancements in detector design and performance optimization, with substantial research efforts focused on Cadmium-Zinc-Telluride (CZT) array detectors targeting capability enhancement. CZT remains an attractive compound semiconductor for radiation detection applications, a preference rooted in its near-ideal bandgap characteristics, elevated density, and superior charge-carrier mobility [59]. Strategic design refinements—including optimization of operational voltage parameters, inter-pixel anode spacing adjustments, and charge cloud transport management—demonstrate measurable improvements in pixellated CZT-SPECT system performance metrics [60]. Such technical progress may yield enhanced spatial resolution and improved energy discrimination capabilities, thereby elevating myocardial perfusion image fidelity and strengthening clinical diagnostic reliability.

### 9.3 Potential Developments in Quantitative MPI

Several potential developments are on the horizon in the field of quantitative MPI, indicating a growing interest in enhancing the accuracy and reproducibility of parameter measurements. Mohy-Ud-Din H. *et al*., proposed a new approach, called physiological clustering, in their study on quantitative myocardial perfusion PET parametric imaging at the voxel level [61]. Consequently, this method substantially reduced noise without significant loss of spatial resolution, enabling enhanced visualization and quantification of MBF and Flow Reserve (MFR). Similar techniques could be developed for CZT-SPECT to improve the accuracy of quantitative analysis.

Moreover, integrating diverse imaging modalities offers novel opportunities to refine prognostic evaluation and guide clinical decision-making to optimize patient outcomes. Consequently, combining CZT-SPECT with complementary imaging techniques such as Cardiac Magnetic Resonance (CMR) or Computed Tomography (CT) could yield synergistic insights by integrating comprehensive functional and anatomical data. Wu D. *et al*., performed a comparative study in Heart Failure (HF) patients, demonstrating that CZT-SPECT provides LV scar quantification, functional assessment, and synchrony evaluation comparable to conventional SPECT [24, 26]. The incorporation of CZT-SPECT with CMR—a modality offering superior spatial resolution for detailed anatomical and functional characterization—could enhance diagnostic precision in myocardial perfusion and cardiac function analysis, thereby improving clinical management strategies.

### 9.4 Long-term Outlook for CZT-SPECT in Cardiovascular Imaging

The long-term prospects for CZT-SPECT in cardiovascular imaging appear promising due to its capacity to deliver functional myocardial perfusion data combined with ongoing technological refinements. It has established itself as an indispensable tool in nuclear cardiology for diagnosing and managing cardiovascular pathologies. Enhanced diagnostic performance, evidenced by studies demonstrating superior sensitivity and marginally increased accuracy over conventional SPECT in detecting angiographically confirmed CAD [23], will likely drive broader clinical adoption.

Additionally, multiple studies demonstrate that CZT-SPECT's potential for reducing radiation dose and employing abbreviated acquisition protocols positions it as a clinically advantageous option, particularly given escalating concerns regarding radiation safety and patient tolerability. The development and iterative refinement of dedicated CZT-SPECT absolute Myocardial Blood Flow (MBF) quantification technology have substantially improved diagnostic efficacy and accuracy in coronary artery disease assessment without elevating patient radiation exposure, while simultaneously advancing prognostic evaluation methodologies. Over time, CZT-SPECT is anticipated to assume an increasingly pivotal role in cardiovascular imaging, enabling precision diagnostics and tailored therapeutic approaches to optimize patient outcomes.

## CONCLUSION

In conclusion, CZT-SPECT plays a vital role in cardiovascular imaging, providing significant benefits over traditional methods, including superior diagnostic accuracy, reduced radiation exposure, and the ability to quantify dynamic myocardial perfusion imaging. Validation against PET and invasive standards has demonstrated excellent diagnostic accuracy, although inter-study variation and device-specific differences remain. The future development of advanced imaging techniques and the integration of multimodal imaging modalities are likely to further enhance the clinical utility of CZT-SPECT. As technology advances, it yields promising results for improving patient outcomes by optimizing diagnostic precision, risk stratification, and tailored therapeutic strategies in managing coronary artery disease. The long-term outlook for CZT-SPECT is optimistic, suggesting that ongoing research enhancements and clinical validation will strengthen its role as a valuable tool for diagnosing and managing cardiovascular conditions effectively.

## Figures and Tables

**Table 1:** Comparison of CZT-SPECT and conventional SPECT.

Features	GE Discovery NM 530c	Spectrum Dynamics D-SPECT	Conventional SPECT
Detector Type	19 Stationary CZT modules with multi-pinhole collimator	9 rotating CZT detector columns	Dual-head rotating NaI detector
Collimator Design	Pinhole collimator (Static, focused on the heart)	Wide-angle rotating collimator per column	Parallel-hole (LEHR,LEGP,et)
Spatial Resolution	~6.5-7mm	~8mm	~10-12mm
Count Activity	~4x Conventional	~6-7x Conventional	Baseline
Dynamic Flow Quantification	Yes (Dynamic MBF/MFR	Yes (Dynamic MBF/MFR)	No
Attenuation Correction	Optional CTAC	No integrated CT	Often available Hybrid SPECT/CT
Artifact Sensitivity	Shift of attenuation artifacts to the lateral wall	Prone to injection site artifacts	Predictable artifacts pattern
Scan Time (Rest/Stress)	~2-5 minutes per acqusition	~2-4 minutes (high speed protocols)	~12-15 minutes or more
Radiation Dose	Low	Low	Higher
Clinical Flexibility	Cardiac only	Cardiac only	Multi-purpose
Equipment Cost	~$375,000–400,000	~$325,000–500,000	~$150,000–250,000
Room Size Requirement	Compact	Medium (upright design, moderate footprint)	Large (rotating dual head + shielded room)
Patient Positioning	Supine or Prone	Upright (Seated)	Supine or Prone

## LIST OF ABBREVIATIONS

**CZT**: Cadmium Zinc Telluride
**SPECT**: Single Photon Emission Computed Tomography
CAD: Coronary Artery Disease
MPI: Myocardial Perfusion Imaging
MBF: Myocardial Blood Flow
MFR: Myocardial Flow Reserve
CFR: Coronary Flow Reserve
CTAC: Computed Tomography Attenuation Correction
BMI: Body Mass Index
**TPD**: Total Perfusion Defect
**RFR**: Relative Flow Reserve
**ROC**: Receiver Operating Characteristics
AUC: Area Under Curve
**MACEs**: Major Adverse Cardiac Events
**INOCA**: Ischemia with Non-Obstructive Coronary Artery
OCAD: Obstructive Coronary Artery Disease
**PET**: Positron Emission Tomography
HF: Heart Failure
**HFpEF**: Heart Failure with Preserved Ejection Fraction
STEMI: ST Elevation Myocardial Infarction
**CMR**: Cardiac Magnetic Resonance
PPV: Positive Predictive Value
**NPV**: Negative Predictive Value
SSS: Summed Stress Score
SRS: Summed Rest Score
SDS: Summed Difference Score
**HR**: Hazard Ratio
**IQR**: Inter Quartile Range
